# Dominance of the zoonotic pathogen *Cryptosporidium meleagridis* in broiler chickens in Guangdong, China, reveals evidence of cross-transmission

**DOI:** 10.1186/s13071-022-05267-x

**Published:** 2022-06-06

**Authors:** Xuhui Lin, Luyao Xin, Meng Qi, Minyu Hou, Shenquan Liao, Nanshan Qi, Juan Li, Minna Lv, Haiming Cai, Junjing Hu, Jianfei Zhang, Xiangbo Ji, Mingfei Sun

**Affiliations:** 1 Zhaoqing/Maoming Branch Center of Guangdong Laboratory for Lingnan Modern Agricultural Science and Technology, Key Laboratory of Livestock Disease Prevention of Guangdong Province, Key Laboratory for prevention and control of Avian Influenza and Other Major Poultry Diseases, Ministry of Agriculture and Rural Affairs; Institute of Animal Health, Guangdong Academy of Agricultural Sciences, Guangzhou, 510640 Guangdong People’s Republic of China; 2 College of Animal Science, Tarim University, Alar, Xinjiang 843300 People’s Republic of China; 3 Lanzhou Veterinary Research Institute, Chinese Academy of Agricultural Sciences, Lanzhou, Gansu 730046 People’s Republic of China; 4 Key Laboratory of Innovation and Utilization of Unconventional Feed Resources, Henan University of Animal Husbandry and Economy, Zhengzhou, 450046 Henan People’s Republic of China

**Keywords:** *Cryptosporidium*, Subtyping, Chicken, Zoonotic, China

## Abstract

**Background:**

*Cryptosporidium* is one of the most prevalent parasites infecting both birds and mammals. To examine the prevalence of *Cryptosporidium* species and evaluate the public health significance of domestic chickens in Guangdong Province, southern China, we analyzed 1001 fecal samples from 43 intensive broiler chicken farms across six distinct geographical regions.

**Methods:**

Individual DNA samples were subjected to nested PCR-based amplification and sequencing of the small subunit of the nuclear ribosomal RNA gene (*SSU* rRNA). Analysis of the 60 kDa glycoprotein gene (*gp*60) was performed to characterize the subtypes of *C. meleagridis*.

**Results:**

The overall prevalence of *Cryptosporidium* was 13.2% (95% CI 11.1–15.3) (24 of 43 farms), with *C. meleagridis* (7.8%), *C. baileyi* (4.8%) and mixed infections (0.6%). Using the *gp*60 gene, three subtype families, IIIb, IIIe and IIIg, were identified, including six subtypes: one novel (IIIgA25G3R1a) and five previously reported (IIIbA23G1R1c, IIIbA24G1R1, IIIbA21G1R1a, IIIeA17G2R1 and IIIeA26G2R1). Within these subtypes, five known subtypes were genetically identical to those identified in humans.

**Conclusions:**

This is the first report of *C. meleagridis* in chickens from Guangdong. The frequent occurrence of *C. meleagridis* in domestic chickens and the common *C. meleagridis* subtypes identified in both humans and chickens is of public health significance. Our study indicates that broiler chickens represent a potential zoonotic risk for the transmission of *Cryptosporidium* in this region.

**Graphical Abstract:**

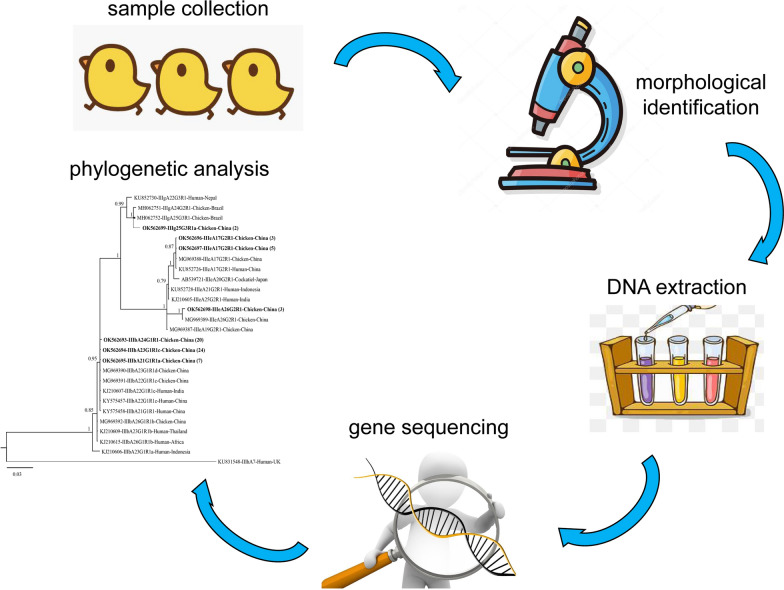

**Supplementary Information:**

The online version contains supplementary material available at 10.1186/s13071-022-05267-x.

## Background

*Cryptosporidium* is a protozoan parasite that infects a wide range of vertebrate hosts, including humans and birds [1]. In birds, *Cryptosporidium* was first found in the order Galliformes, and since then has been reported in more than 30 avian species worldwide [2].

Currently, only five valid species, namely *C. meleagridis*, *C. baileyi*, *C. galli*, *C. avium* and *C. proventriculi*, and at least 15 genotypes including avian genotypes I–II, IV and VI–IX, goose genotypes I–V, black duck genotype, Eurasian woodcock genotype and *C. xiaoi*–like genotype have been documented in a wide range of birds worldwide [3–11]. In addition, mammal-specific *Cryptosporidium* species including *C. hominis*, *C. parvum*, *C. andersoni*, *C. muris* and *C. canis* are rarely detected in birds [12–16], partly because birds ingest oocysts from contaminated food or water and shed oocysts mechanically. *Cryptosporidium baileyi* infection usually occurs in the respiratory system, causing high morbidity and mortality, and *C. meleagridis* infects the gut and is associated with intestinal clinical signs (enteritis and diarrhea), whereas *C. galli* and *C. proventriculi* infect the proventriculus, manifesting symptoms associated with anorexia, weight loss and chronic vomiting [6]. *Cryptosporidium avium* primarily infects the bursa fabricii, but has been described with no pathogenicity [7, 17].

The identification and characterization of species, genotypes and subtypes of *Cryptosporidium* by molecular methods is crucial for the tracing of contamination sources and the assessment of public health importance. Small subunit (*SSU*) rRNA and the 60-kDa glycoprotein (*gp*60) genes are commonly used for determining *Cryptosporidium* species/genotypes and subtyping, respectively [18–20]. Currently, *C. meleagridis* is considered the third most common species infecting humans and the only species that infects both birds and mammals [19]. In China, *C. meleagridis* has been recorded previously in diarrheic children and chickens in Wuhan, Hubei Province; importantly, some *gp*60 *C. meleagridis* subtypes characterized from diarrheic children were shared by chickens in the same location [8, 21]. Recently, avian-specific *Cryptosporidium* species *C. baileyi* pulmonary infection was found in an immunocompetent woman with a benign tumor in Poland [22]. This information highlights the epidemiological importance of avian hosts as significant reservoirs for human cryptosporidiosis, although the extent of cross-species transmission of these zoonotic species remains unclear [4].

Guangdong Province, southern China, is particularly rich in domestic poultry producers. The interaction between humans and domestic poultry poses the potential for zoonotic transmission. However, there are no reports on *Cryptosporidium* isolated from commercial broiler chickens in this region to date, only a study in domestic pigeons [23]. The aim of the present study was to estimate the occurrence and genetic diversity of *Cryptosporidium* species and *C. meleagridis* subtypes, and the public health significance of chickens in intensive farms in Guangdong Province.

## Methods

### Sample collection

**Fig. 1:**
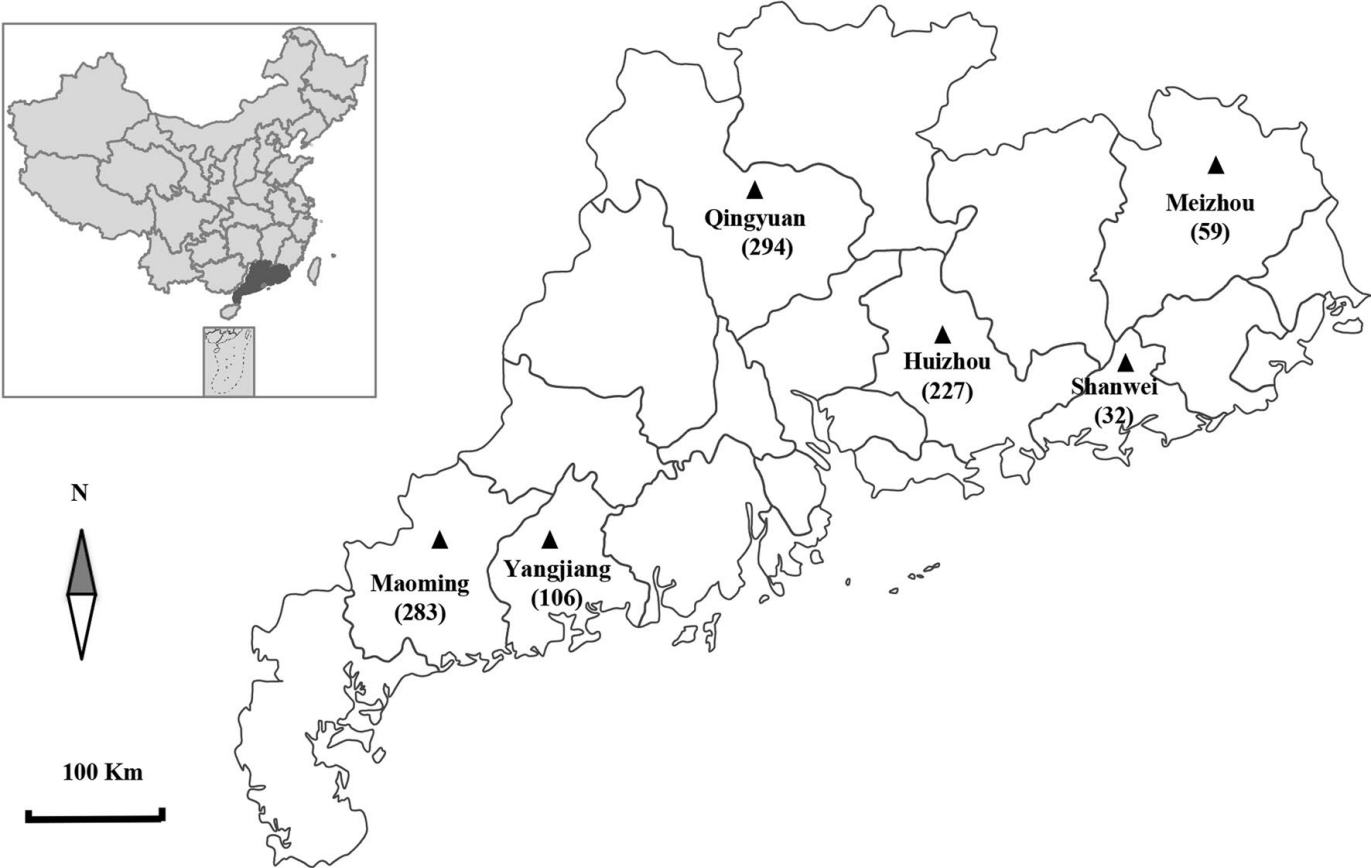
Map of Guangdong showing the locations of the studied cities and number of chicken samples (numbers in parentheses) with geographic distribution

Fresh pooled fecal samples from the floor were randomly collected from broiler chickens (Qing Yuan-ma chickens) from 43 medium-sized to large intensive farms (with 1000–25,000 chickens per farm on average) across six distinct geographical regions (Qingyuan, Maoming, Huizhou, Meizhou, Yangjiang and Shanwei) in Guangdong Province (23°13′S, 113°26′W), China, from June 2020 to March 2021 (Fig. 1; Table 1). Each sample contained 4–5 single fecal deposit droppings from different areas inside the poultry house that were pooled into a single sample. Samples of different consistency and color were chosen on the ground within 1–2 m^2^ to avoid sampling error. Five to 10 samples were collected per farm from broiler flocks comprising 50–100 chickens. A total of 1001 fecal samples of broiler chickens, comprising animals aged < 30 days (*n* = 169), 31–60 days (*n* = 440), 61–90 days (*n* = 348) and > 90 days (*n* = 44), were collected. All samples were collected from apparently healthy flocks. Care was taken to avoid sampling fecal material that had been in contact with the ground. The pooled fecal samples (approximately 50 g) were collected into clean plastic bags, kept in ice boxes and marked with the region, number and date. Samples were then transported immediately to the laboratory and stored at 4 °C. Samples were examined within 24 h after collection.

**Table 1:** Prevalence and species of *Cryptosporidium*. in broiler chickens in Guangdong Province, China

Location	Farms	No. samples	No. positive	% (95% CI)	*Cryptosporidium* species		
					No. of positive samples of *C. baileyi* (%)	No. of positive samples of *C. meleagridis* (%)	No. of positive samples of mixed infection (%)
Qingyuan	14	294	28	9.5 (6.1–12.9)	10 (3.4)	18 (6.1)	–
Maoming	10	283	40	14.1 (10.1–18.2)	6 (2.1)	31 (11.0)	3 (1.1)
Huizhou	9	227	29	12.8 (8.4–17.2)	23 (10.1)	5 (2.2)	1 (0.4)
Yangjiang	4	106	25	23.6 (15.4–31.8)	4 (3.8)	19 (17.9)	2 (1.9)
Meizhou	4	59	7	11.9 (3.4–20.4)	5 (8.5)	2 (3.4)	–
Shanwei	2	32	3	9.4 (0–20.1)	–	3 (9.4)	–
Total		1001	132	13.2 (11.1–15.4)	48 (4.8)	78 (7.8)	6 (0.6)

### DNA extraction and polymerase chain reaction (PCR) amplification

For genomic DNA extraction, approximately 200 mg of fecal samples was suspended in 100 ml of distilled water and centrifuged at 3000×*g* for 10 min. The process was repeated at least three times until the supernatant was clear. Genomic DNA samples were extracted from individual treated materials using the E.Z.N.A._®_ Stool DNA Kit (Omega Bio-tek Inc., Norcross, GA, USA) in accordance with the manufacturer's instructions and then frozen at −20 °C prior to PCR analysis.

Individual DNA samples were subjected to nested PCR-based amplification and sequencing of the small subunit of the nuclear ribosomal RNA gene (*SSU* rRNA, ~ 830 base pairs [bp]) [24]. Subtypes of *C. meleagridis* and mixed-species infections were determined by amplification of the 60 kDa glycoprotein gene (*gp*60; ~ 900 bp) from positive *C. meleagridis* samples and positive *C. baileyi* samples, respectively [20]. PCR was conducted in a 50-μl reaction mixture containing 1× PCR buffer (Takara Shuzo Co., Ltd., Otsu, Japan), 3.0 mM of MgCl_2_, 0.2 mM of each deoxynucleotide triphosphate, 50 pM of each primer, 1 unit of rTaq DNA polymerase (Takara Shuzo Co., Ltd.), 2 μl of DNA sample and 1 μl of bovine serum albumin. Known test-positive (cattle DNA) and test-negative (distilled water) controls were included with each PCR reaction. The amplification products were separated by electrophoresis in 1.5% agarose gels, stained with ethidium bromide and visualized on an ultraviolet (UV) transilluminator.

### Nucleotide sequencing and analysis

All secondary PCR amplicons were sequenced using an ABI PRISM™ 3730xl DNA Analyzer (Applied Biosystems, Foster City, CA, USA) in both directions. Sequences were aligned by the Clustal version 2.1 program and adjusted manually by BioEdit 7.04 software. The adjusted sequences were submitted to a BLAST [Basic Local Alignment Search Tool] search to initially define the species and to further confirm the high similarity with other known sequences of *Cryptosporidium* spp. in the GenBank database.

### Phylogenetic analysis

Phylogenetic analysis was performed using Bayesian inference (BI) and Markov chain Monte Carlo (MCMC) methods in MrBayes version 3.2.6. The best-fit nucleotide substitution model was generalized time-reversible model (GTR + G) determined by ModelTest version 3.7. Based on 1,000,000 generations with four simultaneous tree-building chains, posterior probability values were estimated with trees being saved every 1000th generation. A 50% majority rule consensus tree for each analysis was constructed based on the final 75% of trees generated. To ensure convergence and insensitivity to priors, analyses were run three times by BI. Posterior probabilities of > 0.95 are indicated at all major nodes.

### Statistical analysis

Statistical analysis was performed by chi-square tests, and differences were considered significant when *P* < 0.01 was obtained using SAS version 9.1 (SAS Institute Inc., Cary, NC, USA). Odds ratios (ORs) and 95% confidence intervals (95% CIs) were calculated by SPSS version 22.0 software (IBM Corp., Armonk, NY, USA).

## Results

### Prevalence of *Cryptosporidium*

Of 1001 broiler chicken DNA samples, 132 samples tested positive by PCR amplification of the *SSU* rRNA gene, equating to an overall prevalence of *Cryptosporidium* of 13.2% (95% CI 11.1–15.3%). The PCR-positive chickens were detected on 24 of 43 farms from six geographical regions examined, with prevalence ranging from 4.0% to 62.5% (Additional file 1: Table S1). There was no significant statistical difference in geographical provenance or prevalence among regions (χ^2^ = 14.209, *df* = 5, *P* = 0.014) (Table 1).

### Species and age distribution

*Cryptosporidium* species and genotypes were identified through sequencing of *SSU* amplicons (*n* = 132). This analysis revealed *C. baileyi* in 48 (4.8%) and *C. meleagridis* in 78 (7.8%) of the 132 *SSU* rRNA -positive samples. Six mixed-species infections were also detected. Seven distinct *SSU* rRNA sequences were deposited under GenBank accession numbers OK560460–OK560466. *Cryptosporidium* was detected in all age groups (Table 2), and chickens aged 61–90 days (17.8%) showed a significantly higher infection rate than chickens < 30 days (8.3%), 31–60 days (12.0%) and > 90 days (6.8%) of age (*χ*^2^ = 12.123, *df* = 3, *P* = 0.007). *Cryptosporidium baileyi* was detected in chickens of all age groups (Table 2), and 61–90-day-old chickens tended to have a higher infection rate (8.6%) than other age groups (χ^2^ = 20.600, *df* = 3, *P* = 0.000). *Cryptosporidium meleagridis* was only detected in chickens ≤ 90 days of age, and no statistically significant differences were observed among three age groups (χ^2^ = 0.092, *df* = 2, *P* = 0.955).

**Table 2:** *Cryptosporidium* spp. identified among different age groups of broiler chickens in Guangdong Province, China

Age group	No. samples	No. positive	% (95% CI)	*Cryptosporidium* species		
				No. of positive samples of *C. baileyi* (%)	No. of positive samples of *C. meleagridis* (%)	No. of positive samples of mixed infection (%)
< 30 days	169	14	8.3 (6.7–9.9)	1 (0.6)	13 (7.7)	**–**
31–60 days	440	53	12.0 (11.2–12.8)	14 (3.2)	37 (8.4)	2 (0.5)
61–90 days	348	62	17.8 (16.7–18.9)	30 (8.6)	28 (8.0)	4 (1.1)
> 90 days	44	3	6.8 (2.7–10.9)	3 (6.8)	**–**	–
Total	1001	132	13.2 (11.1–15.4)	48 (4.8)	78 (7.8)	6 (0.6)

### Subtypes of *C. meleagridis*

**Fig. 2:**
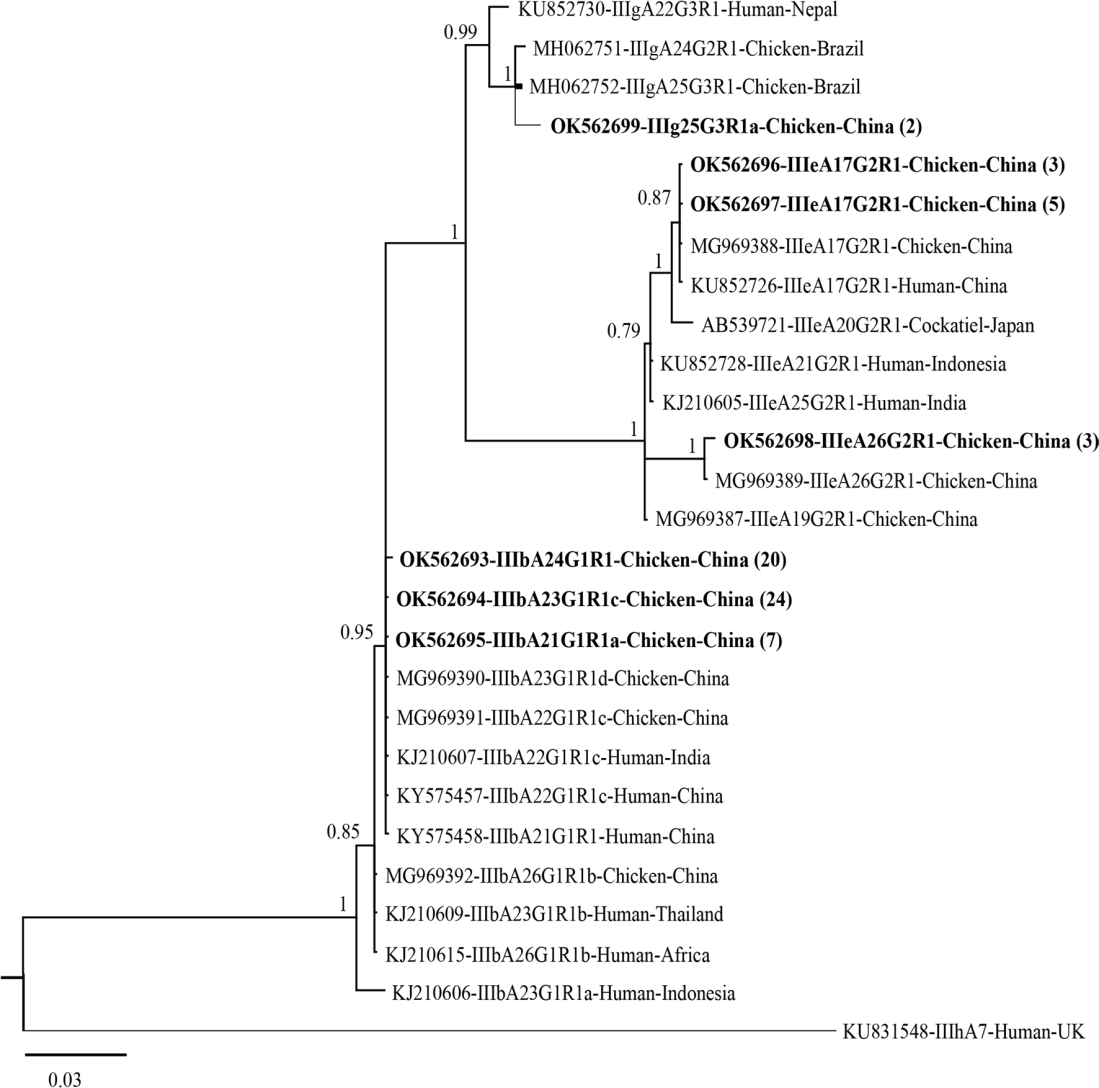
Phylogenetic relationship of the nuclear 60-kDa glycoprotein gene (*pgp60*) of *Cryptosporidium meleagridis* in chickens by Bayesian inference (BI). Posterior probabilities of > 0.95 are indicated at all major nodes. Subtypes tested in this study are labeled after the specimen numbers. The scale bar represents the number of substitutions per site

Among 78 *C. meleagridis*-positive specimens, 64 yielded *gp*60 PCR products of the expected size. Alignment of *gp*60 nucleotide sequences obtained here and references downloaded from the GenBank database revealed the presence of six subtypes, including five known (IIIbA23G1R1c, IIIbA24G1R1, IIIbA21G1R1a, IIIeA17G2R1 and IIIeA26G2R1) and one previously unreported IIIg25G3R1 variant (IIIbA25G3R1a) (GenBank: OK 562,693–562,699). As expected, the phylogenetic tree revealed three distinct clusters, representing three subtype families (IIIb, IIIe and IIIg) (Fig. 2). The most common subtype family, IIIb, was identified in 51 samples, including known subtypes IIIbA23G1R1c (*n* = 24), IIIbA24G1R1 (*n* = 20) and IIIbA21G1R1a (*n* = 7). The second most common subtype family, IIIe, was identified in 11 chicken isolates and comprised three distinct subtypes, two known IIIeA17G2R1 subtypes (*n* = 8) and a known IIIeA26G2R1 subtype (*n* = 3). Finally, for subtype family IIIg, IIIgA25G3R1a was identified in two chicken isolates.

## Discussion

To our knowledge, this is the first report on the presence and prevalence of *Cryptosporidium* in intensively farmed chickens in Guangdong Province, although previous studies have been reported in Hubei, Zhejiang, Henan and Anhui in China [3, 5, 8, 25]. In our study, the overall prevalence of *Cryptosporidium* in chickens (13.2%; 132/1001) was comparable to previous numbers reported for domestic chickens in Brazil (12.6%) [10], China (10.2%) [8] and Syria (9.9%) [26], and higher than Iran (0.5%) [27], Tunisia (4.5%) [28], Jordan (4.8%) [29] and Germany (5.7%) [15], but lower than Brazil (25.6%) [30] and Algeria (34.4%) [4]. Differences in hygiene, management practices, sample origin and detection methods may contribute to these differences in the prevalence of *Cryptosporidium* in poultry flocks.

In addition, *Cryptosporidium* infection in broiler chickens appeared to be age-related. However, unlike the age-related infection pattern whereby the infection rate decreases with increasing age of infected animals in ruminants [31], the highest infection rate of 17.8% was detected in 61–90-day-old broiler chickens, compared with the other age groups (*P* < 0.01) (Table 2). In a previous study in China, chickens ≤ 4 months had the highest infection rate [8], which was partially in agreement with our results. It is worth noting that most broiler chickens are sold by the age of 61–90 days; therefore, oocysts may be disseminated during the process of transfer and new infection may result. These broiler chickens should be considered as important reservoirs of *Cryptosporidium*, although the age-related association should be verified by further research.

*Cryptosporidium meleagridis* and *C. baileyi* were confirmed by molecular characterization of the *SSU* rRNA gene, which was consistent with previous studies reported in farmed and wild birds in China, including chickens, domestic pigeons, quails, ducks, ostriches and white Java sparrows [3, 5, 10, 15, 23, 28, 32, 33]. *Cryptosporidium baileyi*, originally isolated from commercial broiler chickens [34], has a broad range of avian hosts and is considered the predominant avian *Cryptosporidium* species. In China, *C. baileyi* has been reported in a wide variety of birds, including chickens, quails, ostriches, Pekin ducks, domestic pigeons and geese, as well as some pet birds [3, 5, 8, 23, 33, 35]. Recently, *C. baileyi* was also found in an immunocompetent woman with a benign tumor in Poland [22]. Evidence has shown that *C. baileyi* causes respiratory disease and production loss in chickens, causing reduced weight gain in broilers and decreased egg production in laying chickens [36]. One study showed that *C. baileyi* is one cause of Newcastle disease and/or avian influenza vaccination failure in poultry farms [37].

*Cryptosporidium meleagridis* has been detected in various avian hosts, including chickens, turkeys, cockatiels, pigeons and quails, as well as some pet birds [3, 8, 23, 33, 38–40]. *Cryptosporidium meleagridis* has also been frequently detected in humans worldwide, especially in immunocompromised individuals such as neonates and patients with HIV/AIDS [2]. In China, *C. meleagridis* has been detected in diarrheic children in Wuhan [21], HIV-positive patients in Henan [41] and pediatric patients in Shanghai [42]. *Cryptosporidium meleagridis* is an emerging human pathogen and constitutes the third most common human-pathogenic *Cryptosporidium* species after *C. hominis* and *C. parvum* [43–45]. Moreover, molecular studies have revealed that identical *C. meleagridis* subtypes were shared between humans and birds in the same location in Sweden, Peru and China [46], suggesting cross-species transmission of *C. meleagridis* between birds and humans. Chickens may act as a source of infection and a mechanical vector by shedding *C. meleagridis* oocysts into the environment.

Surprisingly, among 132 test-positive chicken fecal samples, *C. baileyi* was detected in the minority (40%) of samples, while *C. meleagridis* was detected in the majority (60%) of samples. The prevalence of *C. meleagridis* in the present study is significantly higher than that of the typical avian species *C. baileyi* [2, 3, 5, 8, 10]. The predominance of *C. meleagridis* among the chicken fecal samples in the present study is consistent with the results of a previous study in poultry in Brazil [47]. Because of the lack of related epidemiological data on cryptosporidiosis in mammals/humans in the investigated areas, the source of infection of domesticated chickens with *C. meleagridis* remains to be elucidated. Whether chickens acquire the infection by contamination of water, feed and/or litter in poultry houses with oocysts from human origin requires further investigation.

To date, at least nine subtype families (IIIa to IIIi) of *C. meleagridis* have been identified by nucleotide sequence analysis of the *gp*60 gene [4, 48, 49]. In the present study, three subtype families of *C. meleagridis*, IIIb, IIIe and IIIg, were detected. Subtypes IIIbA21G1R1a, IIIbA24G1R1, IIIbA23G1R1c, IIIeA17G2R1 and IIIeA26G2R1 have previously been reported sporadically in birds [8, 47], but predominantly in humans, especially those with a travel history to Asia [20, 21, 41, 50]. For example, subtype IIIbA23G1R1c, the predominant subtype found here, had previously been isolated from a Swedish patient with a history of travel to Malaysia, while other variants of this subtype, IIIbA23G1R1a and IIIbA23G1R1b, were reported in patients who had traveled to other developing countries (Indonesia or Thailand) prior to infection [20, 50]. Similarly, subtype IIIbA24G1R1, IIIbA21G1R1a and IIIeA17G2R1 infections had previously been linked to travel to Asian countries (China, Thailand or Vietnam). These reports indicate that foreign travel is a significant risk factor for infection with *C. meleagridis.* Meanwhile, subtype IIIeA26G2R1 identified in chickens in this study was also previously identified in HIV-positive patients in China [41]. This accumulated information suggests the cross-transmission of cryptosporidiosis between chickens and humans in this region. Therefore, to better prevent human cryptosporidiosis, specific management measures are needed on poultry farms, including adherence to an appropriate feeding model as well as strict hygiene and waste management procedures.

## Conclusions

This is the first large-scale molecular study on the occurrence and genetic identity of *Cryptosporidium* in farm-raised chickens in Guangdong Province, China. Two species, *C. meleagridis* and *C. baileyi*, were identified. Five of the six subtypes of *C. meleagridis* detected in this study matched those identified in humans. The dominance of *C. meleagridis* infection among chickens and the detection of zoonotic subtypes IIIbA21G1R1a and IIIbA24G1R1 are indicative of cross-transmission of cryptosporidiosis between chickens and humans. Domestic chickens are of public health significance as potential reservoirs of zoonotic *Cryptosporidium*. Further epidemiological investigations are needed to confirm the source of infection of domesticated chickens with *C. meleagridis*.

## Supplementary Information

**Additional file 1: Table S1**. Samples from 43 intensive chicken farms across six distinct geographical regions (Qingyuan, Maoming, Huizhou, Meizhou, Yangjiang and Shanwei) in Guangdong Province.

## Data Availability

The data supporting the conclusions of this article are included within the article and its additional file. Seven distinct *SSU* rRNA sequences and subtypes sequences of *C. meleagridis* were deposited under GenBank accession numbers OK560460–OK560466 and OK562693–OK562699, respectively.

## Abbreviations

*SSU* rRNA: Small subunit of nuclear ribosomal RNA
*gp*60: 60 KDa glycoprotein
*C.**meleagridis*: *Cryptosporidium* *meleagridis*
*C.**baileyi*: *Cryptosporidium* *baileyi*
