# Supplementary material for: Dominance of the zoonotic pathogen Cryptosporidium meleagridis in broiler chickens in Guangdong, China, reveals evidence of cross-transmission
Source: Parasit Vectors. 2022 Jun 6;15:188. doi: 10.1186/s13071-022-05267-x (PMC9169408; doi:10.1186/s13071-022-05267-x)
Supplement: Supplementary file 1 — Additional file 1: Table S1. Samples from 43 intensive chicken farms across six distinct geographical regions (Qingyuan, Maoming, Huizhou, Meizhou, Yangjiang and Shanwei) in Guangdong Province. [file 13071_2022_5267_MOESM1_ESM.docx]

**Table S**

| Location | Farm | No. of samples/No. of positive samples (%) | *Cryptosporidium* species | | |
| --- | --- | --- | --- | --- | --- |
|  |  |  | *C. baileyi* (%) | *C. meleagridis* (%) | Co-infection (%) |
| Qingyuan | Farm 1-1 | 6/28 (21.4) |  | 6 (21.4), |  |
|  | Farm 1-2 | 0/17 (0) |  |  |  |
|  | Farm 1-3 | 0/17 (0) |  |  |  |
|  | Farm 1-4 | 6/24 (25.0) |  | 6 (25.0) |  |
|  | Farm 1-5 | 0/33 (0) |  |  |  |
|  | Farm 1-6 | 6/22 (27.3) |  | 6 (27.3) |  |
|  | Farm 1-7 | 0/22 (0) |  |  |  |
|  | Farm 1-8 | 0/25 (0) |  |  |  |
|  | Farm 1-9 | 10/16 (62.5) | 10 (62.5) |  |  |
|  | Farm 1-10 | 0/17 (0) |  |  |  |
|  | Farm 1-11 | 0/13 (0) |  |  |  |
|  | Farm 1-12 | 0/17 (0) |  |  |  |
|  | Farm 1-13 | 0/17 (0) |  |  |  |
|  | Farm 1-14 | 0/26 (0) |  |  |  |
|  | Subtotal | 28/294 (9.5) | 10 (3.4) | 18 (6.1) |  |
| Maoming | Farm 2-1 | 11/25 (44.0) |  | 11 (44.0) |  |
|  | Farm 2-2 | 1/25 (4.0) |  | 1 (4.0) |  |
|  | Farm 2-3 | 3/24 (12.5) |  | 3 (12.5) |  |
|  | Farm 2-4 | 11/26 (42.3) |  | 11 (42.3) |  |
|  | Farm 2-5 | 0/28 (0) |  |  |  |
|  | Farm 2-6 | 2/24 (8.3) |  | 2 (8.3) |  |
|  | Farm 2-7 | 7/29 (24.1) | 5 (17.2) |  | 2 (6.9) |
|  | Farm 2-8 | 4/50 (8.0) | 1 (2.0) | 2 (4.0) | 1 (2.0) |
|  | Farm 2-9 | 0/26 (0) |  |  |  |
|  | Farm 2-10 | 1/26 (3.8) |  | 1 (3.8) |  |
|  | Subtotal | 40/283 (14.1) | 6 (2.1) | 31 (11.0) | 3 (1.1) |
| Huizhou | Farm 3-1 | 2/15 (13.3) |  | 2 (13.3) |  |
|  | Farm 3-2 | 0/16 (0) |  |  |  |
|  | Farm 3-3 | 14/31 (45.1) | 13 (41.9) |  | 1 (3.2) |
|  | Farm 3-4 | 0/16 (0) |  |  |  |
|  | Farm 3-5 | 0/15 (0) |  |  |  |
|  | Farm 3-6 | 1/15 (6.7) | 1 (6.7) |  |  |
|  | Farm 3-7 | 3/15 (20.0) | 3 (20.0) |  |  |
|  | Farm 3-8 | 0/15 (0) |  |  |  |
|  | Farm 3-9 | 9/89 (10.1) | 6 (6.7) | 3 (3.4) |  |
|  | Subtotal | 29/227 (12.8) | 23 (10.1) | 5 (2.2) | 1 (0.4) |
| Yangjiang | Farm 4-1 | 6/28 (21.4) |  | 6 (21.4), |  |
|  | Farm 4-2 | 8/27 (29.6) |  | 8 (29.6) |  |
|  | Farm 4-3 | 11/26 (42.3) | 4 (15.4) | 5 (19.2) | 2 (7.7) |
|  | Farm 4-4 | 0/25 (0) |  |  |  |
|  | Subtotal | 25/106 (23.6) | 4 (3.8) | 19 (17.9) | 2 (1.9) |
| Meizhou | Farm 5-1 | 3/16 (18.8) | 1 (6.3) | 2 (12.5) |  |
|  | Farm 5-2 | 2/8 (25.0) | 2 (25.0) |  |  |
|  | Farm 5-3 | 2/17 (11.8) | 2 (11.8) |  |  |
|  | Farm 5-4 | 0/18 (0) |  |  |  |
|  | Subtotal | 7/59 (11.9) | 5 (8.5) | 2 (3.4) |  |
| Shanwei | Farm 6-1 | 0/15 (0) |  |  |  |
|  | Farm 6-2 | 3/17 (17.6) |  | 3 (17.6) |  |
|  | Subtotal | 3/32 (9.4) |  | 3 (9.4) |  |
| Total |  | 132/1001 (13.2) | 48 (4.8) | 78 (7.8) | 6 (0.7) |
